# Supplementary material for: SARS-CoV-2 viral dynamics in non-human primates
Source: PLoS Comput Biol. 2021 Mar 17;17(3):e1008785. doi: 10.1371/journal.pcbi.1008785 (PMC8007039; doi:10.1371/journal.pcbi.1008785)
Supplement: S1 Text — (DOCX) [file pcbi.1008785.s001.docx]

Supplementary Information File 1 : Effects of hydroxychloroquine

Effect of hydroxychloroquine on nasopharyngeal and tracheal viral loads

In our study, 23 macaques were treated with HCQ at various dosing regimen and initiated prophylacticly or at 2 or 5 days post infection. Trough concentrations were measured along the study duration and were used to compute mean plasma concentrations$\tilde{C}$. We evaluated the possibility for HCQ to reduce the infectivity or the viral production by a factor 1- $\eta$ and 1-$\varepsilon$ respectively (see model 1 and 2).

| **Model 1** | **Model 2** |
| --- | --- |
| $\frac{dT_{X}}{dt}=-\beta_{X}(1-\eta)T_{X}V_{X}^{I}$ | $\frac{dT_{X}}{dt}=-\beta_{X}T_{X}V_{X}^{I}$ |
| $\frac{dI_{1,X}}{dt}=\beta_{X}{(1-\eta)T}_{X}{V^{I}}_{X}-kI_{1,X}$ | $\frac{dI_{1,X}}{dt}=\beta_{X}T_{X}{V^{I}}_{X}-kI_{1,X}$ |
| $\frac{dI_{2,X}}{dt}=kI_{1,X}-\delta_{X}I_{2,X}$ | $\frac{dI_{2,X}}{dt}=kI_{1,X}-\delta_{X}I_{2,X}$ |
| $\frac{dV_{X}^{I}}{dt}=p_{X}I_{2,X}\mu-cV_{X}^{I}$ | $\frac{dV_{X}^{I}}{dt}=p_{X}(1-\varepsilon)I_{2,X}\mu-cV_{X}^{I}$ |
| $\frac{dV_{X}^{NI}}{dt}=p_{X}I_{2,X}\left( 1-\mu\right)-cV_{X}^{NI}$ | $\frac{dV_{X}^{NI}}{dt}=p_{X}(1-\varepsilon)I_{2,X}\left( 1-\mu\right)-cV_{X}^{NI}$ |

Where $\eta$ and $\varepsilon$ are the HCQ efficacies and equal to $\frac{\tilde{C}}{\tilde{C}+EC_{50,\beta}}$and $\frac{\tilde{C}}{\tilde{C}+EC_{50,p}}$ respectively.

Both $EC_{50,\beta}$and $EC_{50,p}$ tend to infinity, suggesting an absence of effect of HCQ on limiting the infectivity and the viral production. In order to confirm the absence of antivirtal of HCQ, we correlated the empirical Bayes estimates with HCQ with the concentrations $\tilde{C}$(Fig A and B).


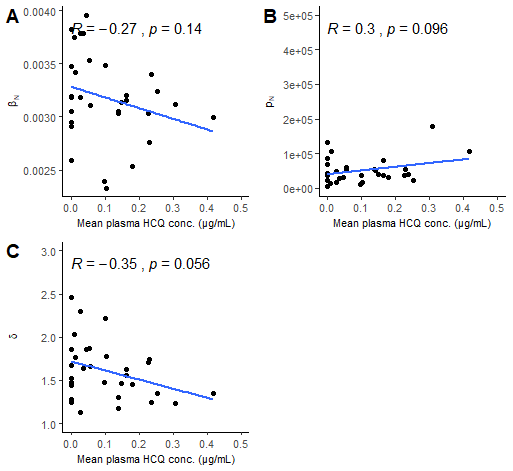


Figure A: Correlation between the empirical Bayes estimates of model 1 and the mean plasma HCQ concentrations supposing an effect on the infectivity $\boldsymbol{\beta}$


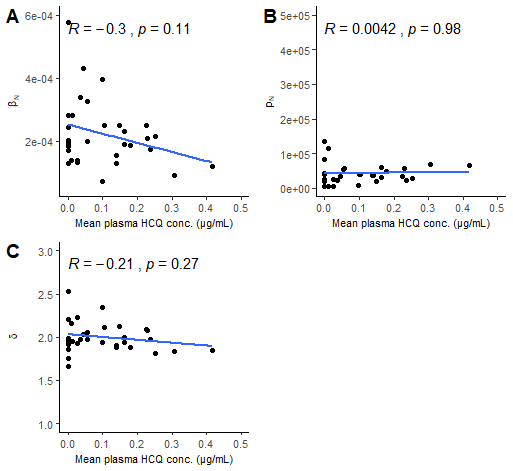


Figure B: Correlation between the empirical Bayes estimates of model 2 and the mean plasma HCQ concentrations supposing an effect on the viral production $\boldsymbol{p}$.
