# Supplementary material for: SARS-CoV-2 viral dynamics in non-human primates
Source: PLoS Comput Biol. 2021 Mar 17;17(3):e1008785. doi: 10.1371/journal.pcbi.1008785 (PMC8007039; doi:10.1371/journal.pcbi.1008785)
Supplement: S3 Text — (DOCX) [file pcbi.1008785.s003.docx]

Supplementary information file 3: Sensitivity analysis

Sensitivity analysis

As the viral clearance *c* and the fraction of infectious viruses *µ* could not be estimated from the data, parameters had to be fixed. However, the values at which those parameters should be fixed remains unknown. Thus, we explored 9 models resulting from the combination of 3 values for *c* and *µ* i.e. {5, 10, 20 d^-1^} and {10^-5^, 10^-4^, 10^-3^} respectively. One can calculate the weight associated to each candidate model based on the BIC and compute the averaged parameter values using model averaging (MA, see Gonçalves et al. 2020). Bayesian information criteria associated to each candidate model are provided in Table A. Model parameter obtained for each candidate model and using MA are provided in Table B and C.

Overall, models showed close BIC within a 5 point range and were barely distinguishable. Parameters estimates are mostly sensitive to the value of µ. Indeed, both infectivity rates $\beta_{T}$ and $\beta_{N}$ decreased with µ increasing. However, all models gave similar R_0_ ~6 and 4 for nasopharynx and trachea, respectively.

**Table A:** Bayesian information criteria of models with different viral clearance c and eclipse phase rate k

| **BIC** | **c = 5 d^-1^** | **c = 10 d^-1^** | **c = 20 d^-1^** |
| --- | --- | --- | --- |
| **µ = 10^-3^** | 1252.0 | 1255.4 | 1255.8 |
| **µ = 10^-4^** | 1252.1 | 1255.1 | 1255.7 |
| **µ = 10^-5^** | 1251.6 | 1254.6 | 1256.2 |

**Table B:** Nasopharyngeal parameter values and associated 95% confidence interval [CI_95%_] across the models and using model averaging. Models resulted from the combination of 3 values for *c* {5, 10, 20 d^-1^} and µ {10^-5^, 10^-4^, 10^-3^}

| c (d^-1^) | µ | $\boldsymbol{\beta}_{\boldsymbol{N}}\boldsymbol{\times}\boldsymbol{1}\boldsymbol{0}^{\boldsymbol{-3}}$ (mL.copies^-1^.d^-1^) | $\boldsymbol{p}_{\boldsymbol{N}}\boldsymbol{\times}\boldsymbol{1}\boldsymbol{0}^{\boldsymbol{4}}$ [CI_95%_]  (copies.d^-1^) | $\boldsymbol{\delta}$ [CI_95%_]  (d^-1^) | $\boldsymbol{R}_{\boldsymbol{0}\boldsymbol{,}\boldsymbol{N}}$ | $\boldsymbol{N}_{\boldsymbol{N}}$ [CI_95%_]  (copies) |
| --- | --- | --- | --- | --- | --- | --- |
| **5** | **µ = 10^-3^** | 0.26  [0.074 - 0.89] | 1.8 [0.36 - 3.2] | 1.9 [1.4 - 2.3] | 9.3 [1.6 - 38] | 9800  [1900 - 18000] |
|  | **µ = 10^-4^** | 1.9  [0.73 - 4.8] | 2 [0.35 - 3.6] | 1.9 [1.5 - 2.2] | 7.2 [1.9 - 19] | 10000  [1900 - 19000] |
|  | **µ = 10^-5^** | 21  [5.3 - 82] | 1.8 [0.44 - 3.2] | 1.9 [1.5 - 2.2] | 7.6 [1.8 - 26] | 9700  [2700 - 16000] |
| **10** | **µ = 10^-3^** | 0.11  [0.051 - 0.26] | 4.5 [1.3 - 7.7] | 1.9 [1.5 - 2.2] | 5.3 [1.9 - 12] | 24000  [7700 - 41000] |
|  | **µ = 10^-4^** | 1.2  [0.48 - 2.8] | 4.8 [1 - 8.6] | 1.9 [1.6 - 2.3] | 5.4 [1.8 - 12] | 25000  [5900 - 43000] |
|  | **µ = 10^-5^** | 12  [5.7 - 25] | 4.6 [1.6 - 7.6] | 1.9 [1.6 - 2.3] | 5.5 [2 - 13] | 24000  [8600 - 38000] |
| **20** | **µ = 10^3^** | 0.11  [0.033 - 0.38] | 9 [1.1 - 17] | 2 [1.5 - 2.4] | 4.6 [1.4 - 11] | 46000  [6600 - 79000] |
|  | **µ = 10^-4^** | 1.1  [0.47 - 2.6] | 9 [2.2 - 16] | 1.9 [1.5 - 2.3] | 4.8 [1.9 - 10] | 47000  [13000 - 78000] |
|  | **µ = 10^-5^** | 12  [2.3 - 67] | 9 [-1.5 - 20] | 1.9 [1.1 - 2.6] | 4.7 [-5.7 - 17] | 48000  [-11000 - 86000] |
| **Model averaging** | | 1.9 [0.078 - 57] | 2.1 [0.43 - 12] | 1.9 [1.5 - 2.3] | 6.8 [1.7 - 27] | 11000  [2500 - 59000] |

**Table C:** Tracheal parameter values and associated 95% confidence interval [CI_95%_] across the models and using model averaging. Models resulted from the combination of 3 values for *c* {5, 10, 20 d^-1^} and µ {10^-5^, 10^-4^, 10^-3^}

| c (d^-1^) | µ | $\boldsymbol{\beta}_{\boldsymbol{T}}\boldsymbol{\times}\boldsymbol{1}\boldsymbol{0}^{\boldsymbol{-3}}$ (mL.copies^-1^.d^-1^) | $\boldsymbol{p}_{\boldsymbol{T}}\boldsymbol{\times}\boldsymbol{1}\boldsymbol{0}^{\boldsymbol{4}}$ [CI_95%_]  (copies.d^-1^) | $\boldsymbol{\delta}$ [CI_95%_]  (d^-1^) | $\boldsymbol{R}_{\boldsymbol{0}\boldsymbol{,}\boldsymbol{T}}$ | $\boldsymbol{N}_{\boldsymbol{T}}$ [CI_95%_]  (copies) |
| --- | --- | --- | --- | --- | --- | --- |
| **5** | **µ = 10^-3^** | 0.41 [0.06 - 2.8] | 0.77 [0.21 - 1.6] | 1.9 [1.4 - 2.3] | 6.2 [0.84 - 36] | 4200  [1200 - 8000] |
|  | **µ = 10^-4^** | 2.7 [0.75 - 10] | 0.84 [0.23 - 1.6] | 1.9 [1.5 - 2.2] | 4.3 [0.99 - 15] | 4500  [1400 - 8200] |
|  | **µ = 10^-5^** | 31 [4.7 - 210] | 0.82 [0.25 - 1.6] | 1.9 [1.5 - 2.2] | 4.9 [0.74 - 27] | 4400  [1500 - 8100] |
| **10** | **µ = 10^-3^** | 0.17  [0.056 - 0.56] | 2.1 [0.81 - 4.3] | 1.9 [1.5 - 2.2] | 3.6 [1.2 - 10] | 11000  [4500 - 22000] |
|  | **µ = 10^-4^** | 1.8 [0.58 - 5.6] | 2.1 [0.69 - 3.9] | 1.9 [1.6 - 2.3] | 3.5 [1 - 9.8] | 11000  [4000 - 19000] |
|  | **µ = 10^-5^** | 18 [6.1 - 53] | 2.2 [0.89 - 4.1] | 1.9 [1.6 - 2.3] | 3.8 [1.2 - 11] | 11000  [5000 - 20000] |
| **20** | **µ = 10^3^** | 0.17 [0.04 - 0.7] | 4.3 [0.66 - 9.9] | 2 [1.5 - 2.4] | 3.2 [0.87 - 9] | 22000  [4000 - 45000] |
|  | **µ = 10^-4^** | 1.7 [0.58 - 4.9] | 4.4 [1.5 - 8.3] | 1.9 [1.5 - 2.3] | 3.5 [1.2 - 9.1] | 23000  [8700 - 42000] |
|  | **µ = 10^-5^** | 20 [2.4 - 150] | 4 [-1.3 - 8.5] | 1.9 [1.1 - 2.6] | 3.2 [-7.5 - 17] | 21000  [-9700 - 39000] |
| **Model averaging** | | 3 [0.084 - 120] | 0.95 [0.26 - 5.7] | 1.9 [1.5 - 2.3] | 4.4 [0.84 - 26] | 5000  [1600 - 29000] |

In humans, it has been reported that 1% of nasopharyngeal epithelial cells express the ACE2 receptor at their surface. However, another protein, TMPRSS2, is also needed to ensure viral entry. Thus, we assumed in the main analysis that 0.1% of target cells co-expressed both proteins. Here we present a comparison of the parameters estimates obtained with 1 and 0.1%.

Assuming 1% leads to a decrease of the value of *p* by a factor 10 (but constant $pT_{0}$). Consequently, this leads to a much lower estimate of $\frac{\mu p}{\delta}$ which becomes <1. In other words, the burst size of infected virus is lower than 1 (see Table below), which is not consistent with the order of magnitude found for R_0_. Thus, we think that proportions of infected cells higher than 0.1% may underestimate the burst size of virus.

Table D: Comparison of parameter estimates obtained assuming 0.1% or 1% of susceptible target cells.

| **% of cells expressing ACE2** | $\boldsymbol{\beta}_{\boldsymbol{N}}$  $\boldsymbol{(\times}\boldsymbol{10}^{\boldsymbol{-3}}\boldsymbol{)}$ | $\boldsymbol{\beta}_{\boldsymbol{T}}$  $\boldsymbol{(\times}\boldsymbol{10}^{\boldsymbol{-3}}\boldsymbol{)}$ | $\boldsymbol{p}_{\boldsymbol{N}}\boldsymbol{T}_{\boldsymbol{N,0}}$  $\boldsymbol{(\times}\boldsymbol{10}^{\boldsymbol{8}}\boldsymbol{)}$ | $\boldsymbol{p}_{\boldsymbol{T}}\boldsymbol{T}_{\boldsymbol{T,0}}$  $\boldsymbol{(\times}\boldsymbol{10}^{\boldsymbol{8}}\boldsymbol{)}$ | $\boldsymbol{\delta}$ | $\boldsymbol{R}_{\boldsymbol{0,N}}$ | $\boldsymbol{R}_{\boldsymbol{0,T}}$ | $\frac{\boldsymbol{\mu}\boldsymbol{p}_{\boldsymbol{N}}}{\boldsymbol{\delta}}$ | $\frac{\boldsymbol{\mu}\boldsymbol{p}_{\boldsymbol{T}}}{\boldsymbol{\delta}}$ |
| --- | --- | --- | --- | --- | --- | --- | --- | --- | --- |
| units | mL/cells/d | mL/cells/d | copies/cells/d | copies/cells/d | 1/d | - | - | copies/cell | copies/cell |
| 0.1% | 1.3 | 2.0 | 9.5 | 3.9 | 1.9 | 5.6 | 3.8 | 2.5 | 1.1 |
| 1% | 1.6 | 2.4 | 8.3 | 3.6 | 1.9 | 6.8 | 4.6 | **0.2** | **0.1** |
