# Supplementary material for: SARS-CoV-2 viral dynamics in non-human primates
Source: PLoS Comput Biol. 2021 Mar 17;17(3):e1008785. doi: 10.1371/journal.pcbi.1008785 (PMC8007039; doi:10.1371/journal.pcbi.1008785)
Supplement: S4 Text — (DOCX) [file pcbi.1008785.s004.docx]

**Supplementary information file 4 : Model building**

As a first step, we considered nasopharynx and trachea parameters as two distinct compartments both described by a target cell limited model (TCL model, see equations below). We then tested whether the virus could migrate from one compartment to the other at a constant first order migration rate g. Parameter g was set to 0 as their was no gain in the BIC and as it did not improve the description of the data (see Table A below).

| $\frac{dT_{N}}{dt}=-\beta_{N}T_{N}V_{N}^{I}$ | $\frac{dT_{T}}{dt}=-\beta_{T}T_{T}V_{T}^{I}$ |  |
| --- | --- | --- |
| $\frac{dI_{1,N}}{dt}=\beta_{N}T_{N}{V^{I}}_{N}-kI_{1,N}$ | $\frac{dI_{1,T}}{dt}=\beta_{T}T_{T}V_{T}^{I} -kI_{1,T}$ |  |
| $\frac{dI_{2,N}}{dt}=kI_{1,N}-\delta_{N}I_{2,N}$ | $\frac{dI_{2,T}}{dt}=kI_{1,T}-\delta_{T}I_{2,T}$ |  |
| $\frac{dV_{N}^{I}}{dt}=p_{N}I_{2,N}\mu-cV_{N}^{I}$ | $\frac{dV_{T}^{I}}{dt}=p_{T}I_{2,T}\mu-cV_{T}^{I}$ |  |
| $\frac{dV_{N}^{NI}}{dt}=p_{N}I_{2,N}\left( 1-\mu\right)-cV_{N}^{NI}$ | $\frac{dV_{T}^{NI}}{dt}=p_{T}I_{2,T}\left( 1-\mu\right)-cV_{T}^{NI}$ |  |

**Table A: Comparison of model with or without migration of viruses between nasopharynx and trachea**

| **Model** | **Description** | $\boldsymbol{\Delta}\boldsymbol{BIC}$ | $\boldsymbol{\sigma}_{\boldsymbol{N}}$ | $\boldsymbol{\sigma}_{\boldsymbol{T}}$ |
| --- | --- | --- | --- | --- |
| Initial model | TCL model  No migration between comp.  $g=0 d^{-1}$  $\delta_{N}\neq\delta_{T}$  $\beta_{N}\neq\beta_{T}$  $p_{N}\neq p_{T}$  Variability on $\beta$, p and $\delta$ | - | 1.21 | 1.06 |
| Migration model | $g$ estimated  $\delta_{N}\neq\delta_{T}$  $\beta_{N}\neq\beta_{T}$  $p_{N}\neq p_{T}$ | +0.2 | 1.19 | 1.05 |

Then we tested the possibility for estimated parameters to be equal in both nasopharynx and trachea and allowed parameters $\beta, p$and $\delta$ to vary between animals. To do this, we used a backward selection procedure and stopped once the BIC did not decrease anymore. Table B below shows comparisons of BIC provided by each model. Overall a model supposing no migration between compartments and $\delta_{N}=\delta_{T}$ best fitted the data.

**Table B: Comparison of models supposing equal parameters between the nasopharynx and the trachea**

| **Model** | **Description** | $\boldsymbol{\Delta}\boldsymbol{BIC}$ | $\boldsymbol{\sigma}_{\boldsymbol{N}}$ | $\boldsymbol{\sigma}_{\boldsymbol{T}}$ |
| --- | --- | --- | --- | --- |
| Initial model | TCL model  No migration between comp.  $g=0 d^{-1}$  $\delta_{N}\neq\delta_{T}$  $\beta_{N}\neq\beta_{T}$  $p_{N}\neq p_{T}$  Variability on $\beta$, p and $\delta$ | - | 1.21 | 1.06 |
| Same $\beta$ | $g=0 d^{-1}$  $\beta_{N}=\beta_{T}$ | +5.4 | 1.27 | 1.08 |
| Same $p$ | $g=0 d^{-1}$  $p_{N}=p_{T}$ | -3.7 | 1.20 | 1.07 |
| **Same** $\boldsymbol{\delta}$ | $\boldsymbol{g=0}\boldsymbol{d}^{\boldsymbol{-1}}$  $\boldsymbol{\delta}_{\boldsymbol{N}}\boldsymbol{=}\boldsymbol{\delta}_{\boldsymbol{T}}$ | **-5.3** | **1.20** | **1.06** |
| Same $\delta$, same $p$ | $g=0 d^{-1}$  $\delta_{N}=\delta_{T}$  $p_{N}=p_{T}$ | -5.2 | 1.19 | 1.07 |
| Same $\delta$, same $\beta$ | $g=0 d^{-1}$  $\delta_{N}=\delta_{T}$  $\beta_{N}=\beta_{T}$ | +4.5 | 1.25 | 1.09 |
| Same $\delta$, same $\beta$, same $p$ | $g=0 d^{-1}$  $\delta_{N}=\delta_{T}$  $\beta_{N}=\beta_{T}$  $p_{N}=p_{T}$ | +2.8 | 1.25 | 1.10 |
