# Supplementary material for: SARS-CoV-2 viral dynamics in non-human primates
Source: PLoS Comput Biol. 2021 Mar 17;17(3):e1008785. doi: 10.1371/journal.pcbi.1008785 (PMC8007039; doi:10.1371/journal.pcbi.1008785)
Supplement: S5 Text — (DOCX) [file pcbi.1008785.s005.docx]

Supplementary Information File 5 : immune response models

Alternative viral dynamic models including immune effectors

We assumed that the immune responses could have various effects in our viral dynamic model and tested whether those alterative models could better describe the data. Based on the cytokines measured in the 31 infected cynomolgus macaques, we explored the correlations between the are under the cytokine curve and the viral load AUC predicted by the model without immune response and used it to build the novel models. Correlations showing a p-value<0.1 were implemented in the model as regressor values instead of describing the cytokine kinetics. However no cytokine showed a significanty correlation with viral load AUC. As those cytokines may not be the only ones involved in the immune response against the virus, we considered generalized immune response models were any immune effector F can interfere with the viral replication. In total, 5 models exploring the immune response during SARS-CoV-2 infection have been explored. Finally we also considered a model were target cells could proliferate. Data fitting was performed using Monolix software (<http://lixoft.com/products/monolix>) and models were compared based on the Bayesian information criteria (BIC): the lower the BIC, the better the model. However, an absolute difference of less than 5 was not sufficient to distinguish the models. For the i^th^ model, we computed the $\Delta BIC$ ($\Delta BIC=BIC_{i}-BIC_{ref}$) and presented them in Table A. Parameter estimates and individual fits of models providing similar or better description of data that the reference model are presented in Table B and Fig A respectively.

Model 1: An immune effector F reduces the infectivity rate

| $\frac{dT_{X}}{dt}=- \boldsymbol{\beta}_{\boldsymbol{X}}\boldsymbol{(1-\varphi}\boldsymbol{F}_{\boldsymbol{X}}\boldsymbol{)}T_{X}V_{X}^{I}$ |  |
| --- | --- |
| $\frac{dI_{1,X}}{dt}=\boldsymbol{\beta}_{\boldsymbol{X}}\boldsymbol{(1-\varphi}\boldsymbol{F}_{\boldsymbol{X}}\boldsymbol{)}T_{X}{VI}_{X}-kI_{1,X}$ |  |
| $\frac{dI_{2,X}}{dt}=kI_{1,N}-\delta_{N}I_{2,N}$ |  |
| $\frac{dV_{X}^{I}}{dt}=p_{X}I_{2,X}\mu-cV_{X}^{I}$ |  |
| $\frac{dV_{X}^{NI}}{dt}=p_{X}I_{2,X}\left( 1-\mu\right)-cV_{X}^{NI}$ |  |
| $\frac{dF}{dt}=qI_{2}-d_{F}F$ | (1) |
|  |  |

Model 2: An immune effector F prevents target cells from infection (irreversible model)

| $\frac{dT_{X}}{dt}=-\beta_{X}T_{X}V_{X}^{I}-\boldsymbol{\varphi}\boldsymbol{F}_{\boldsymbol{X}}\boldsymbol{T}_{\boldsymbol{X}}$  $\frac{\boldsymbol{d}\boldsymbol{R}_{\boldsymbol{X}}}{\boldsymbol{dt}}\boldsymbol{=}\boldsymbol{\varphi}\boldsymbol{F}_{\boldsymbol{X}}\boldsymbol{T}_{\boldsymbol{X}}$ |  |
| --- | --- |
| $\frac{dI_{1,X}}{dt}=\beta_{X}T_{X}{VI}_{X}-kI_{1,X}$ |  |
| $\frac{dI_{2,X}}{dt}=kI_{1,X}-\delta_{X}I_{2,X}$ |  |
| $\frac{dV_{X}^{I}}{dt}=p_{X}I_{2,X}\mu-cV_{X}^{I}$ |  |
| $\frac{dV_{X}^{NI}}{dt}=p_{X}I_{2,X}\left( 1-\mu\right)-cV_{X}^{NI}$ |  |
| $\frac{dF}{dt}=qI_{2}-d_{F}F$ | (2) |

Model 3: An immune effector F prevents target cells from infection (reversible)

| $\frac{dT_{X}}{dt}=-\beta_{X}T_{X}V_{X}^{I}-\boldsymbol{\varphi}\boldsymbol{F}_{\boldsymbol{X}}\boldsymbol{T}_{\boldsymbol{X}}\boldsymbol{+\rho}\boldsymbol{R}_{\boldsymbol{X}}$  $\frac{\boldsymbol{d}\boldsymbol{R}_{\boldsymbol{X}}}{\boldsymbol{dt}}\boldsymbol{=}\boldsymbol{\varphi}\boldsymbol{F}_{\boldsymbol{X}}\boldsymbol{T}_{\boldsymbol{X}}\boldsymbol{+\rho}\boldsymbol{R}_{\boldsymbol{X}}$ |  |
| --- | --- |
| $\frac{dI_{1,X}}{dt}=\beta_{X}T_{X}{VI}_{X}-kI_{1,X}$ |  |
| $\frac{dI_{2,X}}{dt}=kI_{1,X}-\delta_{X}I_{2,X}$ |  |
| $\frac{dV_{X}^{I}}{dt}=p_{X}I_{2,X}\mu-cV_{X}^{I}$ |  |
| $\frac{dV_{X}^{NI}}{dt}=p_{X}I_{2,X}\left( 1-\mu\right)-cV_{X}^{NI}$ |  |
| $\frac{dF}{dt}=qI_{2}-d_{F}F$ | (3) |

Model 4: An immune effector F reduces the loss of infected cells

| $\frac{dT_{X}}{dt}=- \beta_{X}T_{X}V_{X}^{I}$ |  |
| --- | --- |
| $\frac{dI_{1,X}}{dt}=\beta_{X}T_{X}{VI}_{X}-kI_{1,X}$ |  |
| $\frac{dI_{2,X}}{dt}=kI_{1,X}-\delta_{X}I_{2,X}\boldsymbol{-}\frac{\boldsymbol{\varphi}\boldsymbol{F}_{\boldsymbol{X}}\boldsymbol{I}_{\boldsymbol{2,X}}}{\boldsymbol{\theta+}\boldsymbol{F}_{\boldsymbol{X}}}$ |  |
| $\frac{dV_{X}^{I}}{dt}=p_{X}I_{2,X}\mu-cV_{X}^{I}$ |  |
| $\frac{dV_{X}^{NI}}{dt}=p_{X}I_{2,X}\left( 1-\mu\right)-cV_{X}^{NI}$ |  |
| $\frac{dF}{dt}=qI_{2}-d_{F}F$ | (4) |

Model 5: An immune effector F reduces the viral production rate

| $\frac{dT_{X}}{dt}=- \beta_{X}T_{X}V_{X}^{I}$ |  |
| --- | --- |
| $\frac{dI_{1,X}}{dt}=\beta_{X}T_{X}{VI}_{X}-kI_{1,X}$ |  |
| $\frac{dI_{2,X}}{dt}=kI_{1,N}-\delta_{N}I_{2,N}$ |  |
| $\frac{dV_{X}^{I}}{dt}=\boldsymbol{p}_{\boldsymbol{X}}\boldsymbol{(1-\varphi}\boldsymbol{F}_{\boldsymbol{X}}\boldsymbol{)}I_{2,X}\mu-cV_{X}^{I}$ |  |
| $\frac{dV_{X}^{NI}}{dt}=\boldsymbol{p}_{\boldsymbol{X}}\boldsymbol{(1-\varphi}\boldsymbol{F}_{\boldsymbol{X}}\boldsymbol{)}I_{2,X}\left( 1-\mu\right)-cV_{X}^{NI}$ |  |
| $\frac{dF}{dt}=qI_{2}-d_{F}F$ | (5) |

Model 6: Target cells can proliferate

| $\frac{dT_{X}}{dt}=\boldsymbol{r(1-}\frac{\boldsymbol{T}_{\boldsymbol{X}}\boldsymbol{+}\boldsymbol{I}_{\boldsymbol{1,X}}\boldsymbol{+}\boldsymbol{I}_{\boldsymbol{2,X}}}{\boldsymbol{T}_{\boldsymbol{0,X}}}\boldsymbol{)}-\beta_{X}T_{X}V_{X}^{I}$ |  |
| --- | --- |
| $\frac{dI_{1,X}}{dt}=\beta_{X}T_{X}{VI}_{X}-kI_{1,X}$ |  |
| $\frac{dI_{2,X}}{dt}=kI_{1,X}-\delta_{X}I_{2,X}$ |  |
| $\frac{dV_{X}^{I}}{dt}=p_{X}I_{2,X}\mu-cV_{X}^{I}$ |  |
| $\frac{dV_{X}^{NI}}{dt}=p_{X}I_{2,X}\left( 1-\mu\right)-cV_{X}^{NI}$ |  |
| $\frac{dF}{dt}=qI_{2}-d_{F}F$ | (6) |

Models comparison

Model 3 resulted in a reduced BIC of 6 points. We nonetheless decided to keep this model in the supplementary materials for several reasons: 1) from a statistical standpoint the gain in fitting was entirely due to 3 individuals (MF24, MF26 and MF29) and led to more uncertainty in parameter estimates due to increased complexity 2) from a biological standpoint, none of the 6 cytokines measured during the experiments showed a correlation with viral dynamics. Therefore, we think that both biological and statistical considerations do not support the use of this model to fit our data. Parameters estimates and individual fits of model 3 are shown in table S2 and figure S1.

**Table A:** Bayesian information criteria of the alternative tested models

| **Model** | **Description** | $\boldsymbol{\Delta}$**BIC** | **σ_T_** | **σ_N_** |
| --- | --- | --- | --- | --- |
| **Reference model** | Absence of immune response | - | 1.06 | 1.19 |
| **Model 1** | Reduction of infectivity | +3.2 | 1.06 | 1.19 |
| **Model 2** | Emergence of refractory cells that escape from infection | +0.9 | 1.07 | 1.16 |
| **Model 3** | Refractory cells can turn back into target cells | **-6.4** | **1.07** | **1.16** |
| **Model 4** | Reduction of the viral production | +3.5 | 1.06 | 1.20 |
| **Model 5** | Reduction of the loss of infected cells | +3.5 | 1.05 | 1.20 |
| **Model 6** | Proliferation of target cells | +10.6 | 1.06 | 1.19 |

**Table B:** Model 3 - Population parameter estimates

| **Parameters (units)** | **Fixed effects (RSE%)** | **SD of random effects (RSE%)** |
| --- | --- | --- |
| **β_T_ (mL/copie/d)** | 1.3$\times$10^-3^ (86) | 0.4 (58) |
| **β_N_ (mL/copie/d)** | 1.8$\times$10^-4^ (57) |  |
| **p_T_ (copies/d)** | 2.3$\times$10^4^ (130) | 0.9 (75) |
| **p_N_ (copies/d)** | 5.1$\times$10^5^ (84) |  |
| **V_T0_ (copies/mL)** | 1.4$\times$10^8^ (47) | - |
| **V_N0_ (copies/mL)** | 2.9$\times$10^6^ (47) | - |
| **δ (1/d)** | 1.68 (32) | 0.2 (40) |
| **φ** | $9\times$10^-4^ (>100) | - |
| **c (1/d)** | 10 (fixed) | - |
| **k (1/d)** | 3 (fixed) | - |
| **T_N_ (t=0) (cells/mL)** | 1.98$\times$10^4^ (fixed) | - |
| **T_T_ (t=0) (cells/mL)** | 1.88$\times$10^4^ (fixed) |  |
| **σ_T_** | 1.07 (9) | - |
| **σ_N_** | 1.16 (7) | - |

***
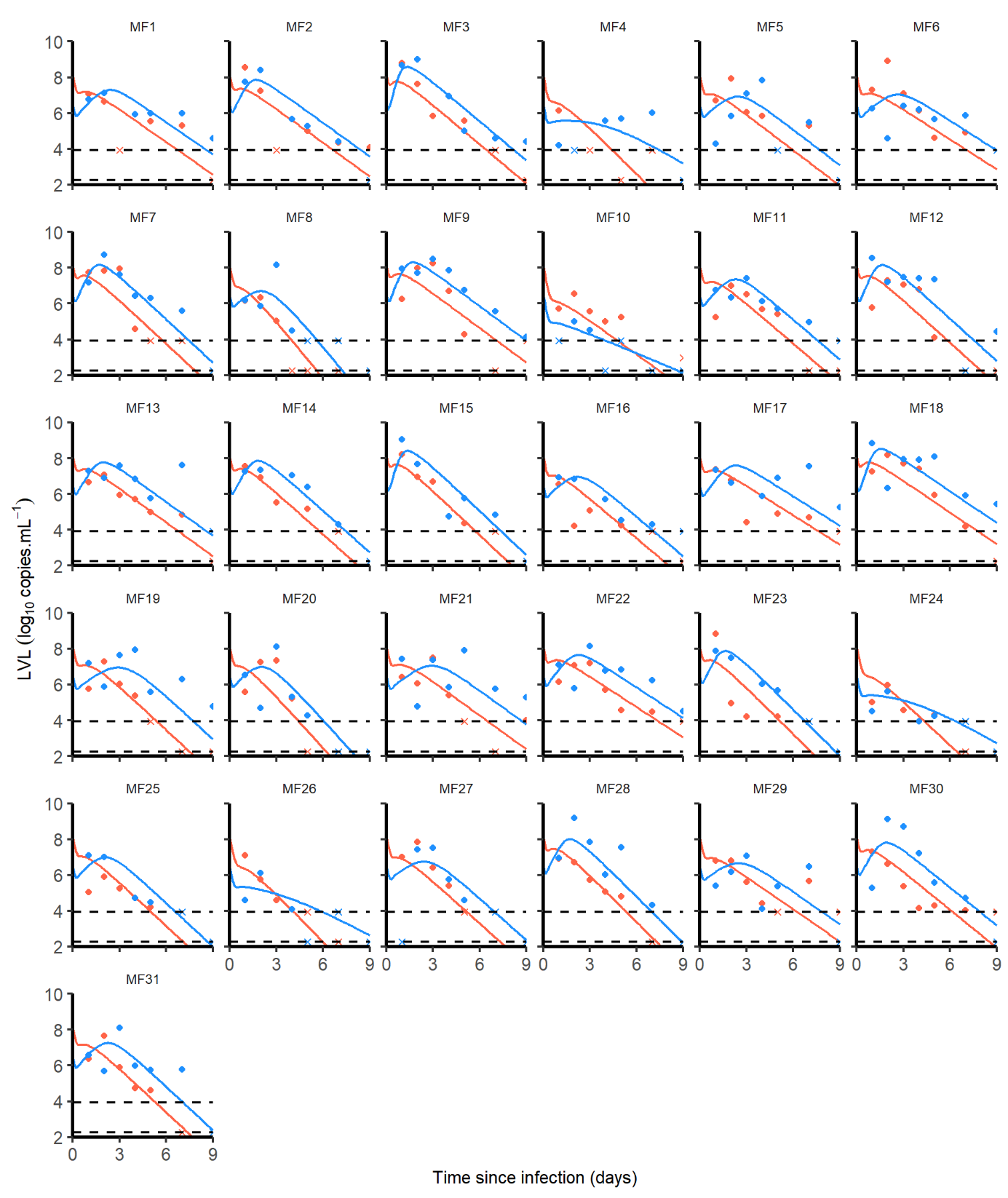
***

Figure A: Nasopharyngeal (blue) and tracheal (red) individual predicted viral loads obtained with model 2 (IFN-⍺ prevents target cells from infection)
