# Supplementary material for: SARS-CoV-2 viral dynamics in non-human primates
Source: PLoS Comput Biol. 2021 Mar 17;17(3):e1008785. doi: 10.1371/journal.pcbi.1008785 (PMC8007039; doi:10.1371/journal.pcbi.1008785)
Supplement: S6 Text — (DOCX) [file pcbi.1008785.s006.docx]

**Supplementary information file 6 : Simulations**

Here, we explored different viral inoculum ranging from 10^4^ to 10^6^ pfu and different drugs mechanism of action and investigated 3 different levels of antiviral efficacy, namely 0, 90 and 99%. Results for a drug blocking viral production or infectious virus production are presented in Figures A and B, respectively. Results for a drug blocking the viral entry are presented in the main text.


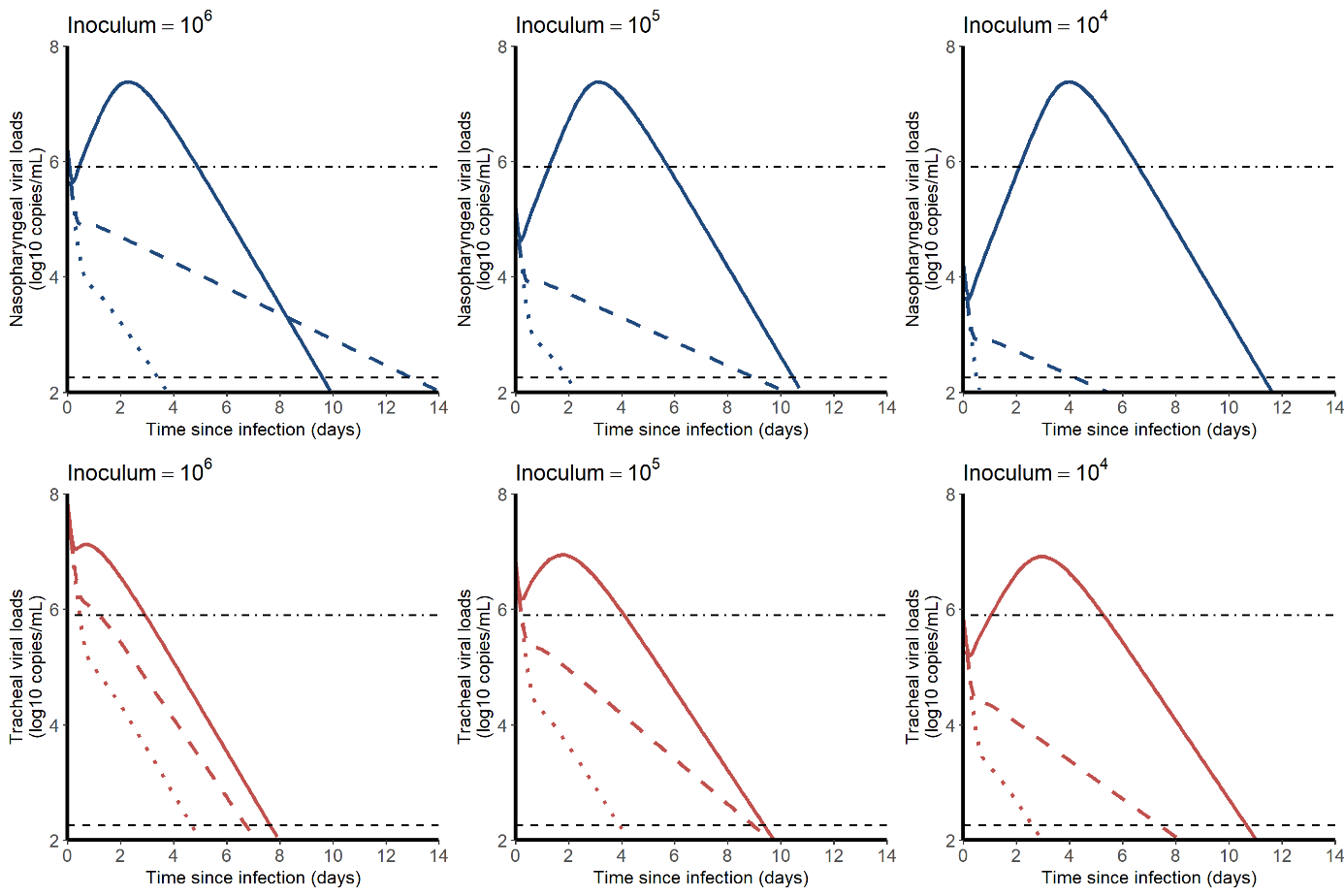


**Figure A**: Median viral kinetic profiles in the nasopharynx (blue) and the trachea (red) according to the inoculum size and the level of an antiviral initiated in prophylaxis and limiting the viral production $p$. Treatment efficacy of 0% (no treatment, solid line), 90% (dashed line) and 99% (dotted lines) were considered.


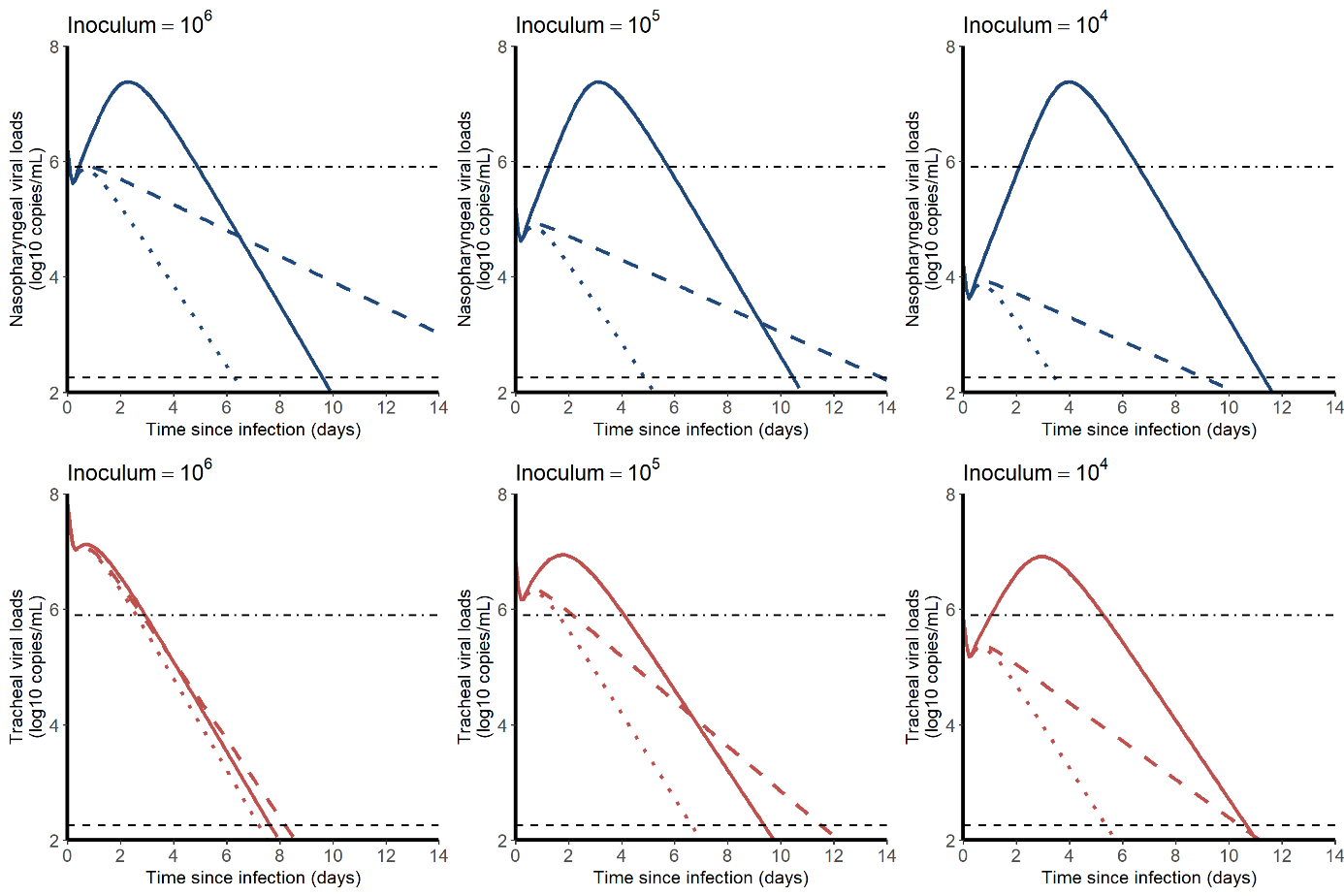


**Figure B**: Median viral kinetic profiles in the nasopharynx (blue) and the trachea (red) according to the inoculum size and the level of an antiviral initiated in prophylaxis and reducing the proportion of infectious viruses $\mu$. Treatment efficacy of 0% (no treatment, solid line), 90% (dashed line) and 99% (dotted lines) were considered.
