# Supplementary material for: SARS-CoV-2 viral dynamics in non-human primates
Source: PLoS Comput Biol. 2021 Mar 17;17(3):e1008785. doi: 10.1371/journal.pcbi.1008785 (PMC8007039; doi:10.1371/journal.pcbi.1008785)
Supplement: Attachment — Submitted filename: 19012021_response_to_reviewers.docx [file pcbi.1008785.s008.docx]

# SARS-CoV-2 viral dynamics in non-human primates

**Response to reviewers**

Dear Editor,

We would like to thank you and the four anonymous reviewers for their interest and thorough review. Please find below our rebuttal letter.

**Points by the Editor:**

**R3: very high challenge dose in the experiments and the complications that introduces for the estimation of R0 would be important to address.**

We agree that high inoculum doses, which are the common rule of animal models, make it more challenging to estimate R_0_. In addition to confidence intervals, we have now improved the discussion on the limits of the experimental model.

**R1: asks you to explore additional mechanisms that might be able to give you a better model fits (point 1).**

We have considerably extended the number of models explored. Per the reviewer’s comment, we have explored immune response models where an immune effector F can reduce the infectivity (Model 1), generate refractory cells that may turn back or not into target cells (Models 2 and 3, respectively), increase the loss of infected cells (Model 4), or reduce the viral production (Model 5). We also challenged the assumption of a target cell limited model (Model 6). All model equations are provided in supplementary information file 3. The refractory model appears to provide a slightly better fit to the data, with a gain in BIC of 6 pts. We nonetheless decided to keep this model in the supplementary materials and not in the main analysis and we motivate this decision by the fact that 1) the gain in fitting was entirely due to 3 individuals (see supplementary file 4) and led to more uncertainty in parameter estimates due to increased complexity 2) none of the cytokines measured showed a correlation with viral dynamics (see Figure 4). Therefore, we think that both statistical and biological considerations do not support the use of this model to fit our data. We added a discussion on this aspect.

**R4: ask you to further justify some model assumptions (point 5).**

As mentioned in the previous comment, we now test more alternatives models. We have also added data on the antibody kinetics to show that the humoral response in unlikely to play a major role in viral clearance.

**In our view, the paper would gain if you justified which mechanisms you chose to include and which potential mechanisms you omitted.**

**Reviewer's Responses to Questions**

**Reviewer #1: To characterize the SARS-CoV-2 nasopharyngeal and tracheal viral load dynamics in 31 cynomolgus macaques, the authors developed a target-cell limited mathematical model for each tissue compartment. The model includes an eclipse phase and the possibility of production of non-infectious viruses. The model is used to estimate basic reproductive ratios for each tissue compartment and other kinetics parameters. The authors also performed correlation tests between immune markers with viral load and explored competing models that include association of viral load and IFN-α levels. They finally made projections of the effect of >90% effective prophylactic interventions. The manuscript is well written and the model is based on a well-known model of virus dynamics. My biggest concern is related to the accuracy of the model fits in capturing the observed behavior, and therefore, the accuracy of parameter estimates and conclusions based on the model.**

**Major comments**

**1. It looks like the viral load observations have different kinetic patterns beyond a viral expansion and clearance, and therefore, the model is not able to capture them. This might be either due to high measurement error or to an additional mechanism that is not in the model (or its extensions using the IFN-α-related models). For example, for the nasopharyngeal observations (blue in Fig 4) there is the possibility of more than one episode/peak (MF6, 13,17,18,19, 21,29) or a plateau/sustained phase where the virus is maintained for several days (MF1, 6, 12 and some of the others mentioned before). In that sense, it is not clear if this phrase in page 5 is completely accurate: “After the peak, both nasopharyngeal and tracheal viral loads rapidly declined exponentially “. It also looks like the model doesn’t capture the nasal dynamics of animals MF4, 5, 10, 26. I suspect that adaptations of this model structure that allows dynamics with a sustained viral load, that may add a couple of parameters could give a significantly lower BIC. I think a larger exploration of models has to be performed to confirm the parameter estimation and conclusions based on the model fits.**

We agree that some viral load data are not perfectly captured by the model. It is however important to realize that these experimental infections reduce the heterogeneity in viral dynamics profiles compared to natural infections, but do not suppress it. This heterogeneity can reveal fundamental biological patterns but can also be simply due to experimental procedures, due to the difficulty to properly assess viral loads in animals.

To address the possibility that important biological mechanisms could be missed by the target cell limited model, we now explore alternative models where an immune effector F is stimulated by antigen and can either reduce the infectivity (Model 1), reduce the number of target cells by putting them into a refractory state (Models 2 and 3, respectively), reduce the viral production (Model 4) or increase the loss of infected cells (Model 5).. Finally, we allow target cells to proliferate to challenge the assumption of cell exhaustion (Model 6).

To ensure model comparison, we used the same structure for the different models, as shown below.

| **Model** | **Description** | **Modifications from the Target Cell limited model** |
| --- | --- | --- |
| Model 1 | Reduction of infectivity | $\frac{dT_{X}}{dt}=- \boldsymbol{\beta}_{\boldsymbol{X}}\boldsymbol{(1-\varphi}\boldsymbol{F}_{\boldsymbol{X}}\boldsymbol{)}T_{X}V_{X}^{I}$  $\frac{dI_{1,X}}{dt}=\boldsymbol{\beta}_{\boldsymbol{X}}\boldsymbol{(1-\varphi}\boldsymbol{F}_{\boldsymbol{X}}\boldsymbol{)}T_{X}{VI}_{X}-kI_{1,X}$  $\frac{\boldsymbol{d}\boldsymbol{F}_{\boldsymbol{X}}}{\boldsymbol{dt}}\boldsymbol{=}\boldsymbol{q}\boldsymbol{I}_{\boldsymbol{2,X}}\boldsymbol{-}\boldsymbol{d}_{\boldsymbol{F}}\boldsymbol{F}_{\boldsymbol{X}}$ |
| Model 2 | Emergence of refractory cells that escape from infection | $\frac{dT_{X}}{dt}=-\beta_{X}T_{X}V_{X}^{I}-\boldsymbol{\varphi}\boldsymbol{F}_{\boldsymbol{X}}\boldsymbol{T}_{\boldsymbol{X}}$  $\frac{\boldsymbol{d}\boldsymbol{R}_{\boldsymbol{X}}}{\boldsymbol{dt}}\boldsymbol{=}\boldsymbol{\varphi}\boldsymbol{F}_{\boldsymbol{X}}\boldsymbol{T}_{\boldsymbol{X}}$  $\frac{\boldsymbol{d}\boldsymbol{F}_{\boldsymbol{X}}}{\boldsymbol{dt}}\boldsymbol{=}\boldsymbol{q}\boldsymbol{I}_{\boldsymbol{2,X}}\boldsymbol{-}\boldsymbol{d}_{\boldsymbol{F}}\boldsymbol{F}_{\boldsymbol{X}}$ |
| Model 3 | Refractory cells can turn back into target cells | $\frac{dT_{X}}{dt}=-\beta_{X}T_{X}V_{X}^{I}-\boldsymbol{\varphi}\boldsymbol{F}_{\boldsymbol{X}}\boldsymbol{T}_{\boldsymbol{X}}\boldsymbol{+\rho}\boldsymbol{R}_{\boldsymbol{X}}$  $\frac{\boldsymbol{d}\boldsymbol{R}_{\boldsymbol{X}}}{\boldsymbol{dt}}\boldsymbol{=}\boldsymbol{\varphi}\boldsymbol{F}_{\boldsymbol{X}}\boldsymbol{T}_{\boldsymbol{X}}\boldsymbol{-\rho}\boldsymbol{R}_{\boldsymbol{X}}$  $\frac{\boldsymbol{d}\boldsymbol{F}_{\boldsymbol{X}}}{\boldsymbol{dt}}\boldsymbol{=}\boldsymbol{q}\boldsymbol{I}_{\boldsymbol{2,X}}\boldsymbol{-}\boldsymbol{d}_{\boldsymbol{F}}\boldsymbol{F}_{\boldsymbol{X}}$ |
| Model 4 | Reduction of the viral production | $\frac{dV_{X}^{I}}{dt}=p_{X}(1-\boldsymbol{\varphi}\boldsymbol{F}_{\boldsymbol{X}})I_{2,X}\mu-cV_{X}^{I}$  $\frac{dV_{X}^{NI}}{dt}=\boldsymbol{p}_{\boldsymbol{X}}\boldsymbol{(1-\varphi}\boldsymbol{F}_{\boldsymbol{X}}\boldsymbol{)}I_{2,X}\left( 1-\mu\right)-cV_{X}^{NI}$  $\frac{\boldsymbol{d}\boldsymbol{F}_{\boldsymbol{X}}}{\boldsymbol{dt}}\boldsymbol{=}\boldsymbol{q}\boldsymbol{I}_{\boldsymbol{2,X}}\boldsymbol{-}\boldsymbol{d}_{\boldsymbol{F}}\boldsymbol{F}_{\boldsymbol{X}}$ |
| Model 5 | Reduction of the loss of infected cells | $\frac{dI_{2,X}}{dt}=kI_{1,X}-\delta_{X}I_{2,X}\boldsymbol{-\varphi}\boldsymbol{F}_{\boldsymbol{X}}\boldsymbol{I}_{\boldsymbol{2,X}}$  $\frac{\boldsymbol{d}\boldsymbol{F}_{\boldsymbol{X}}}{\boldsymbol{dt}}\boldsymbol{=}\boldsymbol{q}\boldsymbol{I}_{\boldsymbol{2,X}}\boldsymbol{-}\boldsymbol{d}_{\boldsymbol{F}}\boldsymbol{F}_{\boldsymbol{X}}$ |
| Model 6 | Proliferation of target cells | $\frac{dT_{X}}{dt}=\boldsymbol{r(1-}\frac{\boldsymbol{T}_{\boldsymbol{X}}\boldsymbol{+}\boldsymbol{I}_{\boldsymbol{1,X}}\boldsymbol{+}\boldsymbol{I}_{\boldsymbol{2,X}}}{\boldsymbol{T}_{\boldsymbol{0,X}}}\boldsymbol{)}-\beta_{X}T_{X}V_{X}^{I}$ |

All but one models led to in increased BIC (see Table below), suggesting that their increased complexity over the target cell limited model are not supported by the data. Model 3 resulted in a reduced BIC of 6 points. We nonetheless decided to keep this model in the supplementary materials for two reasons: 1) from a statistical standpoint the gain in fitting was entirely due to 3 individuals (including MF26 and MF29 that were pointed by the reviewer) and led to more uncertainty in parameter estimates due to increased complexity 2) from a biological standpoint, none of the 6 cytokines measured during the experiments showed a correlation with viral dynamics (see Figure 4). Therefore, we think that both biological and statistical considerations do not support the use of this model to fit our data.

**Table:** Bayesian information criteria of the alternative tested models

| **Model** | **Description** | $\boldsymbol{\Delta}$**BIC** | **σ_T_** | **σ_N_** |
| --- | --- | --- | --- | --- |
| **Reference model** | Absence of immune response | - | 1.06 | 1.19 |
| **Model 1** | Reduction of infectivity | +3.2 | 1.06 | 1.19 |
| **Model 2** | Emergence of refractory cells that escape from infection | +0.9 | 1.07 | 1.16 |
| **Model 3** | Refractory cells can turn back into target cells | **-6.4** | **1.07** | **1.16** |
| **Model 4** | Reduction of the viral production | +3.5 | 1.06 | 1.20 |
| **Model 5** | Reduction of the loss of infected cells | +3.5 | 1.05 | 1.20 |
| **Model 6** | Proliferation of target cells | +10.6 | 1.06 | 1.19 |

**Table:** Model 3 - Population parameter estimates

| **Parameters (units)** | **Fixed effects (RSE%)** | **SD of random effects (RSE%)** |
| --- | --- | --- |
| **β_T_ (mL/copie/d)** | 1.3$\times$10^-3^ (86) | 0.4 (58) |
| **β_N_ (mL/copie/d)** | 1.8$\times$10^-4^ (57) |  |
| **p_T_ (copies/d)** | 2.3$\times$10^4^ (130) | 0.9 (75) |
| **p_N_ (copies/d)** | 5.1$\times$10^5^ (84) |  |
| **V_T0_ (copies/mL)** | 1.4$\times$10^8^ (47) | - |
| **V_N0_ (copies/mL)** | 2.9$\times$10^6^ (47) | - |
| **δ (1/d)** | 1.68 (32) | 0.2 (40) |
| **φ** | $9\times$10^-4^ (>100) | - |
| **c (1/d)** | 10 (fixed) | - |
| **k (1/d)** | 3 (fixed) | - |
| **T_N_ (t=0) (cells/mL)** | 1.98$\times$10^4^ (fixed) | - |
| **T_T_ (t=0) (cells/mL)** | 1.88$\times$10^4^ (fixed) |  |
| **σ_T_** | 1.07 (9) | - |
| **σ_N_** | 1.16 (7) | - |

We modified the results as follows:

*“Among the 30 cytokines tested, 6 greatly varied during the infection and peaked at 2 dpi (namely CCL11, CCL2, IFN-⍺, IL-15, IL-1RA and IL-2) but there was no association between cytokine and viral loads (****Fig. 4****). A model assuming an effect of the immune response in cell protection resulted in a reduced BIC of 6 points. However, the gain in fitting criterion was entirely due to 3 individuals (see* ***supplementary information file 4****) and led to more uncertainty in parameter estimates due to increased complexity (see* ***Table S2****). Moreover none of the cytokines measured during the experiments, including IFN-a, showed a correlation with viral dynamics (****Fig. 5****). Thus, overall, there was no clear evidence in these data that a more complex model improved the understanding of viral dynamics over a simple target cell model.”*

**2. The paper discusses that the viral peak happens about 2 days post-infection. But there is not a discussion of why the model cannot capture the viral peak and that 2-day time.**

We respectfully disagree with the statement that the model cannot capture the viral peak at day 2. As shown in Figure 3, a number of animals (16) have a peak viral load predicted by the model at day 2. We nonetheless agree with the fact that the peak may occur later one and that profiles are heterogeneous, which could be due either to various factors, including the inherent variability of virological assessments relying on nasopharyngeal and tracheal swabs.

**3. There is not a discussion of the reasons to select the values of V0 presented in Table 1. There is only a citation to reference 20 (Long Q-X et al, Nat Med) without giving any reason. I wonder how the model would behave using different values of V0. It is possible that allowing for a lower V0 (or other model structures) can capture the dynamics of some of the nasal viral loads that had low levels or its behavior seems delayed (MF4, 5, 10, 24, 26).**

We apologize for the confusion. The numbers between brackets in Table 1 represent the relative standard error (RSE, %) of the estimated parameters, and do not reference papers. The caption has been clarified.

We agree that the effective inoculum size, V_0_, could vary between animals. This could be captured in our model by allowing V_0_ to have an associated random effect. However, this variability was found <0.1, suggesting that this variability was not supported by the data. To identify this parameter, one would to sample viral load repeatedly in the first hours following infection, which cannot be done experimentally.

**Minor comments**

**4. It was not completely clear if the %RSE in Table 1 was related to the fixed or random effects of the statistical model.**

This has been clarified.

**5. There is a missing “)” at the end of page 6 when describing the estimates of βn.**

This has been modified.

**6. The words “we tested” are repeated in the 4th line in the modeling strategy section in page 17.**

This has been modified.

**Reviewer #2: Goncalves et al. previously reported on their results of a study in non human primates and created a dataset of longitudinal virus load data in the upper respiratory tract (URT) for 31 cynomolgus macaques in response to mild SARS-CoV-2 infections. Here, the authors perform mathematical modeling and, using their data, estimate viral kinetics parameters and hypothesize that potent prophylactic treatment can drive the virus to extinction without prior viral growth. The relatively simple model used in their manuscript was compared against more complicated models including virus migration between nasopharyngeal and tracheal compartment, and cytokine mediated immune responses. These models were not found to explain the data significantly better hinting at a minor role of migration and immune response in the URT during mild SARS-CoV-2 infections.**

**Longitudinal virus load data, in particular data including the pre-symptomatic phase, are extremely rare and hence, despite being limited to the URT and mild infections, the authors study provides an important dataset and estimates for viral kinetics parameters.
However, there are several issues that require attention.**

**1) You report a virus threshold below which viral culture could not be obtained which is a very interesting result. In Fig. 2B, you compare the ratio of TCID50 and viral load with the viral load (“In those for which viral culture could [be] obtained, the ratio of TCID50 to the number of RNA copies ranged between 10-4 to 10-6 (Fig. 2B). “). It is not clear to me what the importance/interpretation of this graph is. Can you please elaborate on this. Are only the ratios important or is the comparison to the viral load important (e.g. correlated/uncorrelated)? Why does this ratio represent an underestimate of the total infectious virus? (p.16 (“the ratio of titers to RNA copies represent an underestimate of the total infectious virus ”))**

The TCID_50_ is the tissue culture infectious dose that generates 50% of the maximal cytopathic effect in Vero E6 cells experiments and is therefore a measure of the infectious viruses present in samples. We have modified the figure 2 to clarify this aspect. Figure 2A shows that 6 log_10_ is the threshold of viral culture *in vitro*, which is a proxy for sample infectiousness. Figure 2B shows moreover that the number of infectious viruses present in samples is directly correlated with the amount of viral RNA. Assuming that 1 TCID_50_ roughly corresponds to 1 infectious virus, the lines drawn in figure 2B shows that the ratio of infectious virus to total RNA is in the order of 10^-5^. However, not all the infectious viruses may grow during the cell culture which makes the measured TCID_50_ a lower bound of infectious viruses. Per your comment we now fixed µ to 10^-4^ and clarified the rationale in methods section: “*TCID_50_ is a proxy for the infectious viruses.* ***Fig. 2B*** *shows that the ratio TCID_50_/total viral loads is 10^-5^ (range 10^-6^ – 10^-4^ ). However, not all the infectious viruses present in the samples may be detected in Vero-E6 cells experiments. Hence, we fixed µ to 10^-4^, the upper bound of the observed ratio of infectious virus.”*

**2) In the model you split the virus into infectious and uninfectious virus. For clarification it may be beneficial to label viral load as total viral load (V^I + V^NI) in your figures.**

This has been modified.

**Furthermore, there are some other issues related to the viral load(s) that require attention (Table 1).**

**- Why is the unit of initial conditions V_T0 and V_N0 copies and not cp/ml? As is, with the given units for beta and T_T(t=0), T_N(t=0), the units in eq. (1) don’t work out.**

Sorry for the confusion, Table I has been modified.

**- If V_T0 and V_N0 are initial conditions for infectious virus only, then label as V^I_T0, V^I_N0. Furthermore, I do not understand how you obtain the values for V_T0 and V_N0, given that (i) the viral inoculation is 10^10 copies (discussion), (ii) 90% (4.5/5ml) of the inoculum are applied to the tracheal and 10% (0.5/5ml) to the nasopharyngeal compartment and (iii) a fraction mu = 10^(-3) of virions is assumed to be infectious.**

$V_{T,0}$and $V_{N,0}$ are the total effective viral loads that initiate the infection, i.e., the sum of infectious and non-infectious viruses. These parameters are estimated with the constraint that $V_{T,0}=9\times V_{N,0}$, which corresponds to the fact that 90% of the viral challenge was inoculated in the trachea (see methods). We estimated V_T,0_ and V_N,0_ to 9.6 10^7^ and 1.1 10^7^ RNA copies corresponding to 8.0 10^7^ and 1.7 10^6^ copies/mL after volume adjustment (Table 1). This corresponds to a total initial viral load of $\sim10^{8}$ RNA copies. This is much lower than the 10^10^ RNA copies used for the viral challenge, and captures the fact that infection is established by only a fraction of the total dose given.

Per your comment, it now reads in results: “*We estimated the effective initial viral load to 1.7*$\times$*10^6^ and 8.0*$\times$*10^7^ copies/mL in nasal and tracheal compartments, respectively, which corresponds to a total inoculum of approximately 10^8^ total RNA, i.e., about 1% of the total injected dose (see methods)”*

**Other minor issues**

**3) In the experiments, is there a reason for splitting the virus inoculation 90% tracheal vs 10% nasopharyngeal? Is this supported by literature of how virus enters the URT?**

This combination of routes is relatively common, and was used internally for various respiratory pathogens (1,2), it has also been used now in the SARS-CoV-2 / NHP studies (3,4). These references have been added to the methods.

**4) (p.5) “After the peak, both nasopharyngeal and tracheal viral loads rapidly declined exponentially, with a median rate of 0.8 log10 copies/mL every day (Fig. 1). The slope in viral decline was more rapid in the trachea than in the nasopharynx, leading to a first measurement below the limit of quantification (LOQ=8514 copies/mL) at 7 and 9 dpi, respectively.
- Is the 0.8 log10 copies/ml the combined median value for nasopharyngeal and tracheal viral loads?**

Per your comment, it now reads: “Afterwards, both nasopharyngeal and tracheal viral loads rapidly declined exponentially, with a similar median rate of 1.9 d^-1^, corresponding to a daily reduction of 0.8 log_10_ copies/mL (Fig. 1). Because the viral load peaked later and higher in in the nasopharynx than in the trachea (7.9 and 7.2 log_10_, respectively), the median time to unquantifiable viral load nonetheless occurred later in the nasopharynx than in the trachea (9 and 7 dpi, respectively)”

**- Given figure 1 it seems to me that the first measurements below LOQ are much earlier than the reported 7 and 9 dpi. Are these average/median values? Please clarify.**

The values reported are medians. Per your comment, Figure 1 has been modified to show the median profile (dashed line).

**5) (p.6) Equation (2): Typo (VI_X instead of V^I_X)**

This has been modified.

**6) (p.7) “Assuming that only 0.1% of cells express ACE2 receptors at their surface (17) “
- According to figure 1b) in ref. (17) approximately 1% of epithelial cells express ACE2. To my understanding figure 1a) from where I assume you extracted the 0.1% shows all cell types. But since you only model epithelial cells I believe you should work with 1%. Since this will change your T_0 estimates, it will probably also have an impact on p and p*T_0.**

We agree with the reviewer that according to reference (17), approximately 1% of epithelial cells express ACE2 in humans and this value was explored in our sensitivity analysis. Using this value mechanically decreases the estimate of p by a factor 10. Consequently, the burst size of infectious virus, $\frac{\mu p}{\delta}$, becomes <1 (see Table below), which is not consistent with the order of magnitude found for R_0_. Thus, we think that proportions of infected cells higher than 0.1% may underestimate the burst size of virus and this is why we keep the value of 0.1% in our main analysis.

| **% of cells expressing ACE2 and TMPRSS2** | $\boldsymbol{\beta}_{\boldsymbol{N}}$  $\boldsymbol{(\times}\boldsymbol{10}^{\boldsymbol{-3}}\boldsymbol{)}$ | $\boldsymbol{\beta}_{\boldsymbol{T}}$  $\boldsymbol{(\times}\boldsymbol{10}^{\boldsymbol{-3}}\boldsymbol{)}$ | $\boldsymbol{p}_{\boldsymbol{N}}\boldsymbol{T}_{\boldsymbol{N,0}}$  $\boldsymbol{(\times}\boldsymbol{10}^{\boldsymbol{8}}\boldsymbol{)}$ | $\boldsymbol{p}_{\boldsymbol{T}}\boldsymbol{T}_{\boldsymbol{T,0}}$  $\boldsymbol{(\times}\boldsymbol{10}^{\boldsymbol{8}}\boldsymbol{)}$ | $\boldsymbol{\delta}$ | $\boldsymbol{R}_{\boldsymbol{0,N}}$ | $\boldsymbol{R}_{\boldsymbol{0,T}}$ | $\frac{\boldsymbol{\mu}\boldsymbol{p}_{\boldsymbol{N}}}{\boldsymbol{\delta}}$ | $\frac{\boldsymbol{\mu}\boldsymbol{p}_{\boldsymbol{T}}}{\boldsymbol{\delta}}$ |
| --- | --- | --- | --- | --- | --- | --- | --- | --- | --- |
| units | mL/cells/d | mL/cells/d | mL/cells/d | mL/cells/d | 1/d | - | - | copies/cell | copies/cell |
| 0.1% | 1.3 | 2.0 | 8.1 | 9.5 | 3.9 | 1.9 | 5.6 | 3.8 | 2.5 |
| 1% | 1.6 | 2.4 | 8.3 | 3.6 | 1.9 | 6.8 | 4.6 | **0.2** | **0.1** |

This is now discussed in the discussion: “*We here estimated the number of alveolar type II cells by analysis of the size of the tissues of one euthanized animal and we assumed that 0.1% of these cells could be target for the virus. Although this value led to more coherent parameter estimates (see supplementary file 2), this is higher than the value of 1% of cells expressing the ACE2 receptor and serine protease TMPRSS2 found in humans (26,27). Specific experiments will be needed to estimate the proportion of target cells in the URT of macaques.*”

**7) ”We found R0,N equal to 5.9 (2.0 – 16) and R0,T equal to 4.0 (1 – 13). Together with the high inoculum in the trachea (see methods), the viral load scarcely increased in the trachea while clearly increased until 3 dpi in the nasopharynx (Fig. 4). One can also derive the viral burst size N corresponding to the number of viruses produced by an infected cell over its lifespan. We found NN = 22,000 (8,100 – 36,000) virions and NT = 10,000 (4,500 – 19,000) virions.”**

**- Please refer to the methods section where you give the formulas for R_0 and N.**

This has been done.

**8) (p.10, Discussion) “we could estimate key parameters of virus pathogenesis, in particular the production rate from infected cells (equal to 1.9 × 10^4^ vs 3.6 × 10^4^ virions/cell/day in tracheal and nasopharyngeal compartments, respectively)”**

**- You say twice (in the results and the methods) that only p*T0 can be identified. For clarification you should state in the discussion that these estimates are obtained for fixed T0. This seems particularly important to me since you claim a certain degree of uncertainty in your T0 estimate on p.11.**

This has been clarified. It now reads in the discussion: “*In this model, p×T0 is the only reliable quantity since the estimate of p depends on the number of susceptible cells.”*

**9) (p.16, Statistical model) How is the function f defined? (How) does it relate to model equations (1)-(5)? Please explain.**

This has been clarified. It now reads: “f is the function describing the total viral loads dynamics over time (V^I^(t)+V^NI^(t))”.

**10) (Supplementary Information file 2) “As the viral clearance c and the eclipse phase rate µ could not be estimated from the data”.**

**- Mu is not the eclipse phase but the fraction of infectious virus.**

This has been modified.

**11) The manuscript should be checked for typos.**

We apologize for the typos. The paper has now been proofread.

**Reviewer #3: In this study Goncalves et al analyze the kinetics of viral load during the entire course of SARS-CoV-2 infection in non-human primates. They describe the dynamics with a mathematical model and estimate model parameters by fitting to data. They also collect longitudinal levels of a panel of cytokines involved in the innate immune response and test for association between these markers and viral load kinetics.**

**There are many very strong and unique aspects of this study, which I think will make it quite valuable for the field. Longitudinal within-host viral load kinetics are still very poorly characterized for COVID-19, since it is very difficult to catch human subjects early in infection (before symptom onset) and sample them frequently. For this reason, the full timecourse of viral load, its variation between individuals, and its association with the disease course are still not clear. Thus, animal models are still very useful. This study represents a very large cohort of animals with very frequent sampling and a large number of biomarkers measured, so it helps fill in some of these gaps. The authors use a mechanistic mathematical model to describe the data, as opposed to just fitting a simple curve, which has the benefit that it then be used to examine how kinetics might change under different parameter perturbations. They use a rigorous statistical method for group-level fitting, which make it easy to then use the model to simulate expected inter-patient variance in kinetics. The availability of so many immunological markers measured alongside viral loads is unique, and the authors use some very nice methods to test for the impact of these markers on viral kinetics. The Discussion section does a very thorough job of describing the many caveats of their work.**

**I think there are two major limitations to this work. I do not think the authors can or should do anything differently to address them, I am simply pointing them out to help with the editorial decision.**

**The first is about the study design in the animal model. It is not clear how much this is helping us understand human infection, because the animals seem to be inoculated with an extraordinarily high dose of virus, and in nearly all animals the entire upslope of the viral load is missing (just like it generally is in human data collected post-symptom onset). We know for many viral infections inoculum size influences disease course, so its possible this high-dose infection model might be qualitatively different than a more realistic infection scenario. And, with the upslope missing, it is nearly impossible to accurately estimate R0 - for infection models within-host or at the population level, it is the early exponential growth phase that provides the information that allows R0 to be an identifiable parameter combination (assuming you separately know/can estimate the lifespan of infected cells). Also, I worry that this initial “overdose” of virus is obscuring some inter-patient variation that exists in natural infection. In humans, there seems to be considerable variation in the time to symptom on set (and therefore likely also in viral load peak), whereas in this model viral load peaks extremely quickly and at a similar time in all animals, so it is unclear how relevant this model is. The second limitation is that there is really not much about this data that motivates the need to apply a mathematical model, and even more specifically the quite complicated model the authors used. I myself am a modeler and do not need to be convinced of the value of models in general … it’s just that here it sort of seems like a model was used just for the sake of using a model. In the paper, Figure 1 is shown, and then the authors launch into a complex model description. But why? When I look at Figure 1, nothing suggests to me you would need or want to apply any sort of mathematical model to this data. It just looks like decay over time. If you are going to write a whole paper about applying a viral dynamics model to data, it would be helpful to provide the reader with some motivation for that! And the model seems relatively complex - again, you need to motivate this. What is the biology you think is going on and why did you decide to capture it this way? How are the nasopharyngeal and tracheal compartments related, physiologically? How would you possibly have enough information to identify migration of cells? Why did you feel the need to include infectious and non-infectious virus? What is the biology behind this and why do you think it’s necessary to explain your data? Why do you think this process is target cell limited? Where did these #s of cells come from? Why are non-productive cells needed? Then looking at Figure 4, honestly the model does not seem to be doing a particularly amazing job explaining any of this data. Mostly, the data seems to be all over the place and barely following a trend. The model seems to be missing the peak in most animals, and not accurately capturing the early kinetics in the few animals for which this info is available.**

We thank you for this stimulating discussion.

First, it is important to keep in mind that these experiments were not designed to study the viral pathogenesis but rather to evaluate the antiviral efficacy of HCQ. In such setting, technical and ethical arguments command to design a protocol that reduces the heterogeneity across animals in order to gain statistical power and reduce the number of animals. Here our primary objective was to assess the antiviral efficacy of hydroxychloroquine (HCQ) and/or azithromycin (detailed in (5)) and this is why animals were infected with a high dose of 10^6^ pfu. Given the total absence of effect of antiviral effect of HCQ, both *in vitro* (6) and *in vivo*, it does not come as a big surprise that our analysis, that relies virological and pharmacological measurements, could not conclude to any antiviral effect of HCQ (see supplementary material for the full analysis).

“Thanks” to the absence of efficacy of HCQ, these data yet provide a unique opportunity to evaluate the determinants of viral kinetics in a large number of animals (N=31) and can be useful to build a model of mild infection (5). This is why we have now considerably extended the number of models tested, and we hope that the parameter values found here could be of interest to better understand the parameters driving viral kinetics of mild infection. Moreover, these results will be important in the perspective of drug evaluation of compounds, such as monoclonal antibodies (mAbs).

We clarified our objectives: “*Here, we used data generated on nonhuman primates (NHP) and treated with hydroxychloroquine (HCQ) ± azithromycine (AZTH) in either pre- or post-exposure prophylaxis* (5) *to develop a mathematical model of SARS-CoV-2 infection. Although, our primary objective was to evaluate the antiviral effects of HCQ, our analysis could not support its existence. However, we used the large quantity of data generated in those experiments (31 cynomolgus macaques with frequent measurements of both nasopharyngeal and tracheal viral loads) to provide estimation of key parameters driving the viral dynamics and evaluate putative immune response mechanisms during the infection.*”

**Despite these concerns, I do still feel like the results of the paper are useful, because we are still very early on in our understanding of this virus and understandably animal models are not yet perfected, and the model the authors use is at least reasonable so can provide a starting point for other work that probes these mechanisms more carefully, and for now can at minimum serve as a calibrated simulation tool for recreating within-host dynamics.**

**A few more specific minor comments, mainly about typos/grammar:**

**Author summary: Typo in these sentences**

**"We found that viral load rapidly declined after peak viral load [despite we found] no association between model parameters and immune markers.”**

This has been modified.

**The within-host reproductive basic reproduction number was estimated to BE 6 and 4 in nasopharynx and trachea suggesting that a prophylactic therapy blocking viral entry or production with 90 efficacy could be sufficient to prevent viral growth and peak viral load.**

This has been modified.

**Introduction:
"This is due to the fact that many studies rely on large transversal analyses with few patients having serial data points or, in contrary, on detailed small series of patients,..”. I think instead of “transversal” you mean “cross-sectional”**

This has been modified.

**Methods:
The paper really does not make sense as it is written, because you jump into Results before explaining the methods. This doesn’t work for a modeling paper. Please include all the model motivation and details that are now at the very end of the paper BEFORE the results section. It is impossible to interpret the results without understanding why you chose the model you did, what all the parameters mean, if you did group level fitting or individual level, which parameters were fixed vs fit, and why were some values fixed and what source did you use to estimate them?**

We thank the reviewer per its comment. We know provide material & methods section before the results.

**I did not understand why it was necessary to include non-infectious virus in the model (I don’t think you are fitting to data that breaks down virus this way), and I did not understand the sentence “Since the ratio of titers to RNA copies represent an underestimate of the total infectious virus and that this ratio ranged from 10-6 to 10-4 (Fig. 2), we fixed μ to 10-3 "**

This has been clarified. It now reads in methods: “*TCID_50_ represents a proxy of the infectious viruses. Fig. 2B shows that the ratio TCID50/total viral loads is 10^-5^ (range 10^-6^ – 10^-4^). However, not all the infectious viruses present in the samples may be detected in Vero-E6 cells experiments. Hence, we fixed µ to 10^-4^, the upper bound of the observed ratio of infectious virus.”*

**Results:**

**"In those for which viral culture could obtained, the ratio of TCID50 to the number of RNA copies ranged between 10-4 to 10-6 (Fig. 2B).” What is TCID50 ? This term was never defined.**

This has been done.

**Figure 1: This figure is pretty useless - it just looks like a pile of points all on top of each other and you can’t follow one animal over time. I think the authors should be able to come up with a better way to show this data. For example, different color lines/points for each animal. And maybe also showing the group mean/median over time.**

Figure 1 aims to show to the reader the high heterogeneity in the data as well as the global trends in both compartments. Per your comment, we now provide the median nasopharyngeal and tracheal profile in addition to the individual dynamics (dashed line).

**Figure 2B: don’t understand what is being shown here. It seems like the same measure (viral load) is being used in both the x and y axis. And again, what is TCID50?**

Per your comment we know present the correlation between TCID_50_ and total viral loads in Figure 2B. The figure shows that the ratio ranged from 10^-6^ to 10^-4^.

**Figure 3: This figure caption is short and non-informative. It should describe the model and what each of the parameters mean.**

This figure has been removed.

**Figure 6: It is obvious from looking at these plots that there is there is no correlation here. I am not sure it is necessary to report all these statistics.**

This has been modified.

**Table 1: What does RSE% mean? Abbreviations should be explained. Table should have another column stating the description of each parameter. The meanings of most of these parameters aren’t explained anywhere - not in the text, or figure caption.**

Per your comment we have clarified the Table caption. It now reads: “*Table 1: Population parameter estimates of the final model described by equation (1) to (5). Numbers in parenthesis are the relative standard error expressed in percentage (RSE%) associated either to the fixed or the standard deviation (SD) of random effects.*”

**Re inoculum size - why is it predicted to take longer for viral clearance with lower innoculum? Would be nice to offer an intuitive explanation, since I found it surprising! Is this because of your assumption that target cell limitation is only mechanism of control? If so, it might be a good experimental test of target cell limited model - try smaller innoculums experimentally and see what happens!**

As long as the infection is target cell limited, a lower initial inoculum would take more time to reach the peak, and therefore could delay the time to resolution. This is not a new finding and we added a reference on this aspect - Li et al., 2014 (7). We have also added in the discussion that additional experiments with different inoculum doses could be helpful to reevaluate alternative models to target cell limited.

**Discussion: Sentences with grammar problems**

**“This hypothesis is supported by the fact that in humans 0.1% of alveolar type II cells expressing the ACE2 receptor, gate for SARS-CoV-2 to enter host cells”**

This has been modified.

**“Such estimate is unknown to our knowledge in cynomolgus macaques”**

This has been modified.

**And I don’t understand what this means: "…we nonetheless note that it leads to coherent parameter estimates verifying the condition of** $\frac{\boldsymbol{\mu p}}{\boldsymbol{\delta}}$**>R0 (i.e., the burst size of the infectious virus is larger than the number of secondary cell infection)"**

R_0_ is the number of cells generated by one infected cell at the beginning of the infection. Thus the burst size of infectious virus, given by $\frac{\boldsymbol{\mu p}}{\boldsymbol{\delta}}$**,** cannot be much lower than R_0_. We explore in the table below the burst size value for different values of the proportion of cells being susceptible (i.e., expressing ACE2 and TMPRSS2). A value of 1% led to a burst size of 0.2, which is much lower than R_0_, which is in the order of 5.

| **% of cells expressing ACE2 and TMPRSS2** | $\boldsymbol{\beta}_{\boldsymbol{N}}$  $\boldsymbol{(\times}\boldsymbol{10}^{\boldsymbol{-3}}\boldsymbol{)}$ | $\boldsymbol{\beta}_{\boldsymbol{T}}$  $\boldsymbol{(\times}\boldsymbol{10}^{\boldsymbol{-3}}\boldsymbol{)}$ | $\boldsymbol{p}_{\boldsymbol{N}}\boldsymbol{T}_{\boldsymbol{0,N}}$  $\boldsymbol{(\times}\boldsymbol{10}^{\boldsymbol{8}}\boldsymbol{)}$ | $\boldsymbol{p}_{\boldsymbol{T}}\boldsymbol{T}_{\boldsymbol{0,T}}$  $\boldsymbol{(\times}\boldsymbol{10}^{\boldsymbol{8}}\boldsymbol{)}$ | $\boldsymbol{\delta}$ | $\boldsymbol{R}_{\boldsymbol{0,N}}$ | $\boldsymbol{R}_{\boldsymbol{0,T}}$ | $\frac{\boldsymbol{\mu}\boldsymbol{p}_{\boldsymbol{N}}}{\boldsymbol{\delta}}$ | $\frac{\boldsymbol{\mu}\boldsymbol{p}_{\boldsymbol{T}}}{\boldsymbol{\delta}}$ |
| --- | --- | --- | --- | --- | --- | --- | --- | --- | --- |
| **units** | **mL/cells/d** | **mL/cells/d** | **mL/cells/d** | **mL/cells/d** | **1/d** | **-** | **-** | **copies/cell** | **copies/cell** |
| 0.1% | 1.3 | 2.0 | 9.5 | 3.9 | 1.9 | 5.6 | 3.8 | 2.5 | 9.5 |
| 1% | 1.6 | 2.4 | 8.3 | 3.6 | 1.9 | 6.8 | 4.6 | **0.2** | **0.1** |

Hence, the model assuming 0.1% of target cells was favored. This is now discussed in the discussion: “*We here estimated the number of alveolar type II cells by analysis of the size of the tissues of one euthanized animal and we assumed that 0.1% of these cells could be target for the virus. Although this value led to more coherent parameter estimates (see supplementary file 2), this is higher than the value of 1% of cells expressing the ACE2 receptor and serine protease TMPRSS2 found in humans (26,27). Specific experiments will be needed to estimate the proportion of target cells in the URT of macaques.”*

**Final note: When submitting a paper for review, the authors should include figures in the main text near where they are first cited, with the figure captions directly below the images. It is very inconsiderate to reviewers to expect them to jump back and forth between different sections of the paper to try to interpret figures. Also, some of the images are bad quality, which makes it very difficult to interpret the results.**

Although we agree that this is not convenient, the Editor does not allow figures in the main text but only as separate files. Per your comment, we have increased the figures quality as much as possible given the Editor requirements.

**Reviewer #4: The authors use a simple within-host model to describe the kinetics of SARS-CoV-2. Although the model itself is not new, the authors make use of both in vivo and in vitro data to estimate important parameters (e.g. R0 and the lifespan of infected cells) and distinguish likely infectious virus from non-infectious virus. Overall the manuscript is clear and well-written though lacking in detail in a few places.**

**Major points:**

**1. The authors assume HCQ reduces viral production, but are there other possible mechanisms (e.g. reducing the transmission rate, Supplementary File 1, Fig S1), and if so, does accounting for these support the assumption of no effect of HCQ on viral dynamics?**

We agree with the reviewer that HCQ could act by other mechanisms through the induction of the immune response and leading to a reduction of the infectivity. Per your comment, we added a model where HCQ could reduce the infectivity of the virus, but results are unchanged,which is not surprising given the similarity in viral dynamics across the treatment groups. Results are presented in supplementary file 1.

**2. Results page 10: The authors focus on drugs with 90 and 99% efficacy and identify 90% as a target efficacy for future drug development that would prevent infection. But were lower efficacies investigated? For example, was 90% the lowest efficacy at which infection was prevented? Even if so, drugs with lower efficacies may still limit infection to a significant enough degree that it would be beneficial to include them as potential targets.**

Given that R_0_ < 10, an efficacy of 90% would be sufficient to reduce the viral loads in both compartments. This value is in line with what has been found in humans and corresponds to the efficacy needed to prevent the infection if treatment is initiated prior to the exposure (8,9). We have clarified the discussion as follows: “*Given our estimate of R_0_, a 90% effective treatment should be able to prevent virus growth and would maintain the viral load levels below 10^6^ copies/mL at all times, making the chance of detecting infectious virus very limited, consistent with our modeling predictions in humans (*(9)*, submitted).”*

**3. Discussion: It would be informative to discuss how the parameter estimates compare to those of other within-host models for SARS-CoV-2 (at least in human studies, there have been a wide range of reported R0 and infected cell lifespan estimates).**

Estimating R_0_ requires access to repeated viral load data during its upslope. These data are difficult to access, since they would require to catch patients prior to symptom onset. Some data start to be published (10) but there has not been a modeling analysis to our knowledge. In a previous publication we estimated R_0_ to 8 but in a limited number of individuals. In Goyal et al. the value could not be directly compared as the model is not target cell limited.

**4. Methods: To improve reproducibility, more information on the fitting procedure is needed, for example, on the backward selection procedure, and the implementation in Monolix (e.g. initial estimates). These could be included as supplementary material. I would encourage the authors to share their code for complete reproducibility.**

Per your comment, we now provide a supplementary file with comparisons of all models tested during the backward selection as well as the model code and dataset.

**5. Given that infection is mild here it seems perhaps unlikely that infection is limited by depletion of all susceptible cells which - despite their assumed low abundance — would likely still involve considerable damage and inflammation. It seems at least equally plausible that the infection is limited by innate immune mechanisms or NAbs. Could you justify the choice of a target cell limited model a priori? Does the choice of model influence the key conclusions? Some discussion of this issue would strengthen the paper.**

Alternative models including immune response models have been explored but none of them showed a better description of the data compared to the target cell limited model (see Supplementary material 3 and reviewer #1 comment 1). Regarding, the neutralizing antibodies, we had access to the dosing of S1, S2 and N195 antibodies by LIPS (luciferase immunoprecipitation system) in all 31 animals. As shown below, the percentage of animals with detectable antibodies remained null until day 14, day by which all the animals had already undetectable viral loads. Hence, effect of antibodies on the reduction of viral loads is unlikely and we did not further explore this effect. Per your comment, this is now discussed in the discussion: “*In addition, none of the animals had detectable antibodies until day 7, and only 25% had detectable antibodies by day 14, suggesting that the humoral response played a minor role in viral clearance.*”.

**Minor points:**

**1. p7 - p x T0 is referred to as total viral production — this seems a little misleading as it’s never the case that all T0 targets are simultaneously infected. p15 - you say “First, the term p×T0 is the only identifiable quantity in our model, “… this is a typo - I presume you’re saying that only this combination is identifiable, not p and T0 individually.**

This has been modified.

**2. Results of prophylaxis treatment, page 10: The authors investigate different levels of viral inoculum, but it is not clear how these levels, quoted in PFU, were translated to V(0), quoted in copies (e.g. Table 1), to perform the simulations?**

The inoculum of 10^6^ pfu corresponded to 8.0x10^7^ copies/mL and 1.7x10^6^ copies/mL in the trachea and the nasopharynx. When simulating the dynamics after the inoculation of 10^5^ and 10^4^ pfu, we supposed that the corresponding V_0_ decreased linearly by a factor 10 and 100, respectively. It now reads in methods section: “*We explored lower viral inoculum of 10^5^ and 10^4^ pfu by simulating with lower initial viral loads (10 and 100 fold lower, respectively) as well as different drugs mechanisms of action (blocking viral production, viral entry, or infectious virus production), with different levels of antiviral efficacy.*”

**3. Model comparisons: In model comparison tables (e.g. Supplementary FiIe 3, Table S1) it would be easier for the viewer to distinguish between model support by quoting the difference in BIC between each model and the reference model (rather than the absolute BIC value, which is meaningless)**

This has been modified.

**4. Fig 4: Some macaques, nasal virus load persists for some time and there is even a suggestion of a second peak (e.g. MF13, MF17, MF29). Can the authors speculate as to why this may be (in some cases these later measurements appear to be above the threshold for obtaining infectious virus)?**

The later peaks observed in some individuals could be due some immune response mechanisms or proliferation of target cells. Those models were investigated and did not further improved the description of the data (see reviewer #1 comment 1 and supplementary file 3).

**5. Fig 7: It would be useful to the reader to add a horizontal line to each panel marking the threshold below which infectious virus could not be obtained in the in vitro experiments.**

This has been done.

**6. Fig 7 bottom row panels (tracheal viral loads): As I understand things, the solid curves represent dynamics for a drug with 0% efficacy (i.e. no drug) and should be the same regardless of mode of action. In other words, for each viral inoculum panel in Fig 7, the solid curve should be the same as that in the corresponding viral inoculum panel in Figures S1 and S2 (Supplementary File 4). However, this does not seem to be the case (e.g. the solid curves in Fig 7 appear to peak later than those in Figs S1 and S2)?**

We thank the reviewer for noticing this discrepancy. This has been modified.

**More generally, the authors should show somewhere in the main text or supplementary material how each mode of action is incorporated into the model equations.**

Per your comment, we now provide in supplementary file 1 all the equations related to the modes of action of HCQ on both the infectivity and the viral production.

**7. General comment: Did the authors find any differences in dynamics between male and female macaques? As differences have been reported with respect to human infection, it would be interesting to know if any differences are apparent in the macaque data.**

Per your comment, we checked the differences in the observed peak, time to peak, viral AUC and time to undetectability. Overall, we found no differences between animals. Important to note that the animals were randomly assigned in sex balanced experimental groups (see (5)).

**References**

1.	Kobasa D, Jones SM, Shinya K, Kash JC, Copps J, Ebihara H, et al. Aberrant innate immune response in lethal infection of macaques with the 1918 influenza virus. Nature. 18 janv 2007;445(7125):319‑23.

2.	Cillóniz C, Shinya K, Peng X, Korth MJ, Proll SC, Aicher LD, et al. Lethal influenza virus infection in macaques is associated with early dysregulation of inflammatory related genes. PLoS Pathog. oct 2009;5(10):e1000604.

3.	Rockx B, Kuiken T, Herfst S, Bestebroer T, Lamers MM, Munnink BBO, et al. Comparative pathogenesis of COVID-19, MERS, and SARS in a nonhuman primate model. Science. 29 mai 2020;368(6494):1012‑5.

4.	Mercado NB, Zahn R, Wegmann F, Loos C, Chandrashekar A, Yu J, et al. Single-shot Ad26 vaccine protects against SARS-CoV-2 in rhesus macaques. Nature. oct 2020;586(7830):583‑8.

5.	Maisonnasse P, Guedj J, Contreras V, Behillil S, Solas C, Marlin R, et al. Hydroxychloroquine in the treatment and prophylaxis of SARS-CoV-2 infection in non- human primates. Nature [Internet]. 6 mai 2020 [cité 3 juin 2020]; Disponible sur: https://www.researchsquare.com/article/rs-27223/v1

6.	Pizzorno A, Padey B, Julien T, Trouillet-Assant S, Traversier A, Errazuriz-Cerda E, et al. Characterization and treatment of SARS-CoV-2 in nasal and bronchial human airway epithelia. 2 avr 2020 [cité 7 mai 2020]; Disponible sur: http://biorxiv.org/lookup/doi/10.1101/2020.03.31.017889

7.	Li Y, Handel A. Modeling inoculum dose dependent patterns of acute virus infections. Journal of Theoretical Biology. avr 2014;347:63‑73.

8.	Gonçalves A, Bertrand J, Ke R, Comets E, de Lamballerie X, Malvy D, et al. Timing of antiviral treatment initiation is critical to reduce SARS-CoV-2 viral load. medRxiv [Internet]. 21 juin 2020 [cité 27 juill 2020]; Disponible sur: https://www.ncbi.nlm.nih.gov/pmc/articles/PMC7276997/

9.	Czuppon P, Débarre F, Gonçalves A, Tenaillon O, Perelson AS, Guedj J, et al. Predicted success of prophylactic antiviral therapy to block or delay SARS-CoV-2 infection depends on the targeted mechanism. 12 mai 2020; Disponible sur: http://medrxiv.org/lookup/doi/10.1101/2020.05.07.20092965

10.	Kissler SM, Fauver JR, Mack C, Tai C, Shiue KY, Kalinich CC, et al. Viral dynamics of SARS-CoV-2 infection and the predictive value of repeat testing. medRxiv. 23 oct 2020;2020.10.21.20217042.
